# Completeness of reporting in abstracts of randomized controlled trials in subscription and open access journals: cross-sectional study

**DOI:** 10.1186/s13063-019-3781-x

**Published:** 2019-12-02

**Authors:** Iva Jerčić Martinić-Cezar, Ana Marušić

**Affiliations:** 1 Center for Transfusion Medicine, University Hospital Center Split, Split, Croatia; 2 Department of Research in Biomedicine and Health, University of Split School of Medicine, Šoltanska 2, 21000 Split, Croatia

**Keywords:** Reporting guidelines, Randomized controlled trial, CONSORT for Abstracts, Open access publishing, Subscription journals

## Abstract

**Background:**

Open access (OA) journals are becoming a publication standard for health research, but it is not clear how they differ from traditional subscription journals in the quality of research reporting. We assessed the completeness of results reporting in abstracts of randomized controlled trials (RCTs) published in these journals.

**Methods:**

We used the Consolidated Standards of Reporting Trials Checklist for Abstracts (CONSORT-A) to assess the completeness of reporting in abstracts of parallel-design RCTs published in subscription journals (*n* = 149; *New England Journal of Medicine*, *Journal of the American Medical Association*, *Annals of Internal Medicine*, and *Lancet*) and OA journals (*n* = 119; BioMedCentral series, PLoS journals) in 2016 and 2017.

**Results:**

Abstracts in subscription journals completely reported 79% (95% confidence interval [CI], 77–81%) of 16 CONSORT-A items, compared with 65% (95% CI, 63–67%) of these items in abstracts from OA journals (*P* < 0.001, chi-square test). The median number of completely reported CONSORT-A items was 13 (95% CI, 12–13) in subscription journal articles and 11 (95% CI, 10–11) in OA journal articles. Subscription journal articles had significantly more complete reporting than OA journal articles for nine CONSORT-A items and did not differ in reporting for items trial design, outcome, randomization, blinding (masking), recruitment, and conclusions. OA journals were better than subscription journals in reporting randomized study design in the title.

**Conclusion:**

Abstracts of randomized controlled trials published in subscription medical journals have greater completeness of reporting than abstracts published in OA journals. OA journals should take appropriate measures to ensure that published articles contain adequate detail to facilitate understanding and quality appraisal of research reports about RCTs.

## Background

Randomized controlled trials (RCTs) are considered the best way to compare therapeutic or preventive interventions in medicine [1]. Clear, transparent, and complete reporting of RCTs is necessary for their use in practice and in health evidence synthesis [2, 3]. It is important that presentations of RCTs in abstracts are also complete and clear, because trial validity and applicability can then be quickly assessed. Also, in some settings, such as in developing countries, an abstract may be the only source of information for health professionals because of limited access to the full texts, and the use of abstracts as sole sources of information may adversely influence healthcare decisions [3]. To improve the quality of reporting of RCT abstracts, an extension of the Consolidated Standards of Reporting Trials (CONSORT) statement was developed in 2008 [2, 3]. The Consolidated Standards of Reporting Trials Checklist for Abstracts (CONSORT-A) statement specifies a minimum set of items that authors should include in the abstract of an RCT [3]. So far, evidence shows poor adherence in general and specialty medical journals [4–7].

Currently, more than half of the studies indexed in the largest biomedical bibliographical database Medline are in open access (OA) [8]. It was claimed that the advent of OA journals would lead to the erosion of scientific quality control. This opinion was based on the assumption that the OA publishers would take over an increasing part of the publishing industry and would not provide the same level of rigorous peer review as traditional subscription publishers, which would result in a decline in the quality of scholarly publishing [9]. However, there is evidence that the overall quality of OA journal publishing is comparable to that in traditional subscription publishing [10, 11]. The aim of this study was to assess the completeness of results reporting in abstracts of RCTs published in traditional subscription journals (members of the International Committee of Medical Journal Editors [ICMJE] [12]) and in OA journals (two oldest journal consortia: Public Library of Science [PLoS] journals and BioMedCentral [BMC] series journals).

## Methods

This cross-sectional study included all abstracts of articles about RCTs published in four subscription journals (*New England Journal of Medicine* [*NEJM*], *JAMA* [*Journal of the American Medical Association*], *Annals of Internal Medicine* [*AIM*], and *The Lancet*) and two collections of OA journals (*BMC* series journals and *PLoS* journals) from January 2016 to December 2017. *BMJ* (*British Medical Journal*), which is an ICMJE member, was not included in this group, because it has a combination of OA and subscription publishing options and was previously a fully OA journal [13].

Two researchers independently screened the articles for inclusion of articles describing the basic study design for which CONSORT was developed: randomized, double-blind, two-group parallel design. The following study designs were thus excluded: crossover trials, cluster trials, factorial studies, pragmatic studies, superiority trials, noninferiority trials, megatrials, sequential trials, open-label studies, nonblinded studies, single-blind studies, pilot studies, secondary analysis of primary trials, and combined studies (RCT plus other study designs). The literature search, outlined in Additional file 1, was undertaken in the MEDLINE database using the OvidSP interface.

The completeness of reporting in the abstracts was independently assessed by two researchers using the CONSORT-A checklist with 16 items. We did not include the item “authors” (i.e., “contact details for the corresponding author”), because this item is specific to conference abstracts [3]. The completeness of reporting was presented as the percentage of articles in two journal groups reporting the individual items, the average percentage and 95% confidence interval (CI) of reported items for the two journal groups, the median number (95%CI) of reported items for each article group, and the mean difference (95% CI) between abstracts published in 2016 and 2017. The results were compared using the chi-square test, *t* test, and Mann-Whitney test (MedCalc Statistical Software, Ostend, Belgium).

## Results

**Fig. 1:**
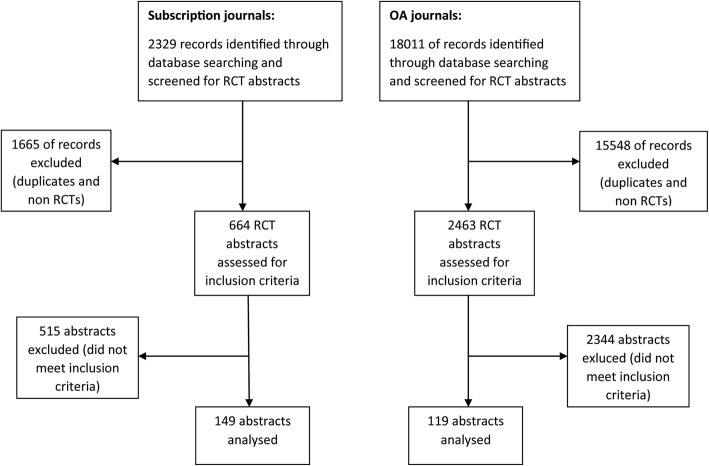
Selection of abstracts of published randomized controlled trials for analysis

A MEDLINE search retrieved 2329 abstracts published in the subscription journals and 18,011 abstracts published in the OA journals. After screening, 149 abstracts published in the subscription journals (63 [42%] in *NEJM*, 44 [30%] in *Lancet*, 36 [24%] in *JAMA*, 6 [4%] in *AIM*) and 119 abstracts published in the OA journals (56 [47%] in *BMC* series journals and 63 [53%] in *PLoS* journals) remained for analysis (Fig. 1).

Articles in subscription journals had, on average, 79% (95% CI, 77–81%) completely reported items of total 16 items of the CONSORT-A, compared with 65% (95% CI, 63–67%) for articles in OA journals (*P* < 0.001, chi-square test). The abstracts in subscription journals had a median of 13 (95% CI, 12–13) reported items, and OA journals had a median of 11 (95% CI, 10–11) reported items, of a total of 16 CONSORT-A items (*P* < 0.001, Mann-Whitney test).

Table 1 presents the completeness of reporting of individual CONSORT-A items. Recruitment was the most completely reported item for both journal groups. The item randomization in subscription journals and the item funding in OA journals had the least complete reporting. Only two abstracts in OA journals contained information about funding (one from pharmaceutical company and one from noncommercial sources). Among the abstracts in subscription journals that reported funding (112 of 149 total [75%]), 63% were from pharmaceutical companies, 30% were from noncommercial sources, and 7% were from both sources. At the level of individual CONSORT-A checklist items, the abstracts in subscription journals had significantly more complete reporting than those in OA journals for all items except trial design, outcome, randomization, blinding (masking), recruitment, and conclusions, where there was no difference in reporting. Abstracts in articles published in OA journals had significantly more complete reporting than subscription journals for the title item. This was due to the fact that in one of the subscription journals, *NEJM*, the title of the article indicated the study type in only 2 (3%) of 63 abstracts, which represented 42% of the subscription journal article sample. The results for individual journals are presented in Additional file 2.

**Table 1:** Number and percentage of articles (95% confidence interval for percentage) published in subscription or open access journals in 2016–2017 satisfying individual items on the CONSORT-A checklist

Item	Subscription journals (*n* = 149)	OA journals (*n* = 119)	*P* value (χ^2^ test)
1. Title	86 (58%; 49–66%)	95 (80%; 72–87%)	0.001
2. Trial design	141 (95%; 89–97%)	110 (92%; 86–96%)	0.465
Methods			
3. Participants	149 (100%; 97–100%)	113 (95%; 89–98%)	0.006
4. Interventions	149 (100%; 97–100%)	115 (97%; 91–99%)	0.024
5. Objective	132 (89%; 82–93%)	115 (97%; 91–99%)	0.015
6. Outcome	146 (98%; 94–99%)	111 (93%; 87–97%)	0.054
7. Randomization	46 (31%; 24–39%)	46 (39%; 30–48%)	0.183
8. Blinding (masking)	130 (87%; 81–92%)	97 (82%; 73–88%)	0.196
Results			
9. Number randomized	87 (58%; 50–66%)	53 (45%; 36–54%)	0.024
10. Recruitment	149 (100%; 97–100%)	117 (98%; 93–100%)	0.113
11. Number analyzed	71 (48%; 39–56%)	29 (24%; 17–33%)	0.001
12. Outcome	64 (43%; 35–5%)	15 (13%; 7–20%)	< 0.001
13. Harms	114 (77%; 69–83%)	33 (28%; 20–37%)	< 0.001
14. Conclusions	149 (100%; 97–100%)	116 (97%; 92–99%)	0.052
15. Trial registration	149 (100%; 97–100%)	100 (84%; 76–90%)	< 0.001
16. Funding	112 (75%; 68–81%)	2 (2%; 0–7%)	< 0.001

*CONSORT-A* Consolidated Standards of Reporting Trials for Abstracts, *OA* open access

There was no difference in the completeness of reporting between the two publication years analyzed in our study: 2016 (total *n* = 145 abstracts) and 2017 (total *n* = 123 abstracts): 2016–2017 mean difference (MD), − 4.07; 95% CI, − 8.11% to − 0.02% for subscription journals (*P* = 0.0487); and MD, 3.99; 95% CI, − 0.32% to 8.31% (*P* = 0.0692) for OA journals.

## Discussion

We found that the abstracts of articles on RCTs published in subscription medical journals had better reporting completeness according to CONSORT-A than abstracts published in OA journals. There was no difference in the completeness of reporting between 2016 and 2017 in both journal groups, indicating that this was a real phenomenon reflecting a standard practice and not a temporal fluctuation. It is important to keep in mind that all journals included in our study state explicitly that they follow reporting standards as set in reporting guidelines, such as CONSORT.

The limitations of the study include the fact that we included only well-known traditional and OA journals, so that the results may represent best practices and underestimate adequate reporting in health journals. We had very strict inclusion criteria and restricted the comparison only to two-group, double-blind, parallel trial design, which left out many other trial study designs. The CONSORT statement was originally created for the “standard” two-group parallel design, and CONSORT-A was developed for the original CONSORT checklist; therefore, we decided to take this basic design as the inclusion criterion, because it is possible that journals from the two groups in our study may differ in the types and complexity of the trials they publish, which may represent a significant bias. The journals in our study were predominantly general medical journals and published in developed countries, so they may not be fully representative of the general population of medical journals. We also assessed the completeness of results reporting in the abstracts and not the full text. We decided to include only abstracts because they are available in bibliographical databases, which are often the primary route of access to information for many health professionals [14]. This is especially true for settings where health professionals have limited access to the full texts and read only abstracts of journal articles. In such cases, inadequate reporting in abstracts could seriously mislead a reader regarding interpretation of the trial findings [15, 16]. Although an article abstract should be a clear and accurate reflection of what is included in the article, several studies have highlighted problems in the accuracy and quality of abstracts [17–20].

The greatest differences between the OA and subscription journals were in adequate descriptions of outcomes and harms, which were more often reported in subscription than in OA journals. In general, underreporting of selective reporting of outcomes is a serious problem, particularly when harms are not reported [21–23]. Although subscription journals published this information at least twice as often as OA journals did, the level of reporting of outcomes and harms is below desirable complete reporting (43% of abstracts fully describing outcomes and 77% describing harms). This underreporting has serious consequences because it may impede the interpretation of the benefit-to-risk relationship.

Both OA and subscription journals adhered to the registration policy; all abstracts in subscription journals had trial registration numbers compared with 84% in OA journals. Only 2% of the abstracts in OA journals reported funding, compared with 75% in subscription journals. It is difficult to draw conclusions about these differences in funding reporting, because only 2 abstracts of 119 in OA journals contained this information. However, it is clear that subscription journals practice greater transparency in reporting funding in abstracts of clinical trials.

A possible explanation for the observed differences in trial-reporting completeness in abstracts in our study is that subscription journals have more resources than OA journals, but this is most probably not the case for the journals included in our study. The representatives of OA journals in our study were well-established PLoS journals and BMC series journals: PLoS journals were started with a US$9 million grant [24], and BMC series journals are published by the Springer Nature group, one of the largest scientific publishers [25]. Article processing fees are up to US$3000 for *PLoS Medicine* [26] and US$3170 for *BMC Medicine* [27]. It is difficult to compare the revenues of OA journals with those of major ICMJE subscription journals in our study because their revenues are not generally known [28], but there is no reason to believe that OA journals included in our study did not have resources for implementing reporting guidelines and ensuring the completeness of published abstracts. All journals included in the study are selective and have high volumes of submissions, with an acceptance rate of approximately 5% for subscription journals [29–32]. In the OA group, *PLoS Medicine* has a 3% acceptance rate [33], whereas BMC series journals have a higher acceptance rate, 45–55%, with some of its journals having acceptance rates below 10% [34].

On the one hand, it can be argued that authors are responsible for completeness of reporting of their studies, including in the abstract. On the other hand, it has been shown that editorial interventions after manuscript acceptance significantly improve the quality of abstracts [35]. Journals are thus well-positioned to ensure that reporting guidelines are followed. They can also help their authors by endorsing tools that have been developed to help authors improve the completeness of their reports, such as the web writing tool based on CONSORT [36]. Recent developments in this field include the Penelope decision-making tool, developed by Penelope Research and the Enhancing the Quality and Transparency of Health Research (EQUATOR) Network [37]. The tool was tested in four BMC series journals in 2016, where it is presented to authors as an embedded element in the manuscript submission system [37]. On the one hand, this indicates that OA journals are open to innovations for better reporting and that they may be more advanced than subscription journals in that respect. On the other hand, subscription journals traditionally offer full editorial support to authors to improve their manuscripts for publication, including abstracts [38, 39].

## Conclusion

Our study showed that reporting of RCTs in article abstracts is less complete in OA journals than in subscription journals. OA journals should address this problem and demonstrate that they can publish high-quality articles. After the launch of the cOAlition S initiative to provide full and immediate open access to research publications a reality by 2020 in Europe, OA journals may gain even more importance in publishing [40]. In order to fulfill their expected role, OA journals publishing health research should take appropriate measures to ensure that published articles contain adequate detail to facilitate understanding and quality appraisal of research reports about RCTs.

## Supplementary information

**Additional file 1.** Search strategies for open access and subscription journals.

**Additional file 2.** Reporting of CONSORT for Abstracts for individual journals.

## Data Availability

The data are available from the corresponding author. The datasets generated and analyzed during the current study will be available in the Croatian Digital Academic Archives and Repositories (https://dabar.srce.hr/repozitoriji).

## Abbreviations

*AIM*: *Annals of Internal Medicine*
BMC: BioMedCentral
*BMJ*: *British Medical Journal*
CI: Confidence interval
CONSORT-A: Consolidated Standards of Reporting Trials Checklist for Abstracts
ICMJE: International Committee of Medical Journal Editors
*JAMA*: *Journal of the American Medical Association*
MD: Mean difference
*NEJM*: *New England Journal of Medicine*
OA: Open access
PLoS: Public Library of Science
RCT: Randomized controlled trial
