# Supplementary material for: Completeness of reporting in abstracts of randomized controlled trials in subscription and open access journals: cross-sectional study
Source: Trials. 2019 Dec 2;20:669. doi: 10.1186/s13063-019-3781-x (PMC6889688; doi:10.1186/s13063-019-3781-x)
Supplement: Supplementary file 1 — Additional file 1. Search strategies for open access and subscription journals. [file 13063_2019_3781_MOESM1_ESM.docx]

**Appendix 1. Search strategies to identify randomized controlled trials in subscription and open access journals**

**1. Search strategy for RCT studies published in 2016 and 2017 in open access journals:**

1 (plos biology or plos computational biology or plos currents or plos genetics or "plos medicine public library of science" or plos neglected tropical diseases electronic resource or plos one electronic resource or plos pathogens).jn.

2 (bmc biochemistry or bmc bioinformatics or bmc biophysics or bmc biotechnology or bmc cell biology or bmc developmental biology or bmc ecology or bmc evolutionary biology or bmc genetics or bmc genomics or bmc immunology or bmc microbiology or bmc molecular biology or bmc neuroscience or bmc physiology or bmc plant biology or bmc structural biology or bmc systems biology or bmc veterinary research electronic resource).jn.

3 (bmc anesthesiology or bmc cancer or bmc cardiovascular disorders or bmc clinical pathology or bmc complementary & alternative medicine or bmc dermatology or bmc ear nose & throat disorders or bmc emergency medicine or bmc endocrine disorders or bmc family practice or bmc gastroenterology or bmc geriatrics or bmc health services research or bmc hematology or bmc infectious diseases or bmc international health & human rights or bmc medical education or bmc medical ethics or bmc medical genetics or bmc medical genomics electronic resource or bmc medical imaging or bmc medical informatics & decision making or bmc medical research methodology or bmc musculoskeletal disorders or bmc nephrology or bmc neurology or bmc nursing or bmc nutrition or bmc obesity or bmc ophthalmology or bmc oral health or bmc palliative care or bmc pediatrics or bmc pharmacology & toxicology or bmc pregnancy & childbirth or bmc psychiatry or bmc psychology or bmc public health or bmc pulmonary medicine or "bmc sports science medicine and rehabilitation" or bmc surgery or bmc urology or bmc womens health).jn. (50798)

4 1 or 2 or 3 (237003)

5 randomized controlled trial.pt. (137289)

6 controlled clinical trial.pt. (11139)

7 randomized.ab. (175064)

8 placebo.ab. (60676)

9 drug therapy.fs. (465980)

10 randomly.ab. (113002)

11 trial.ab. (189782)

12 groups.ab. (642271)

13 or/5-12 (1279859)

14 exp animals/ not humans.sh. (706663)

15 13 not 14 (1151238)

16 4 and 15 (48629)

17 limit 16 to yr="2016" (9678)

2. **Search strategy for RCT studies published in 2017 in open access journals:**

1 (plos biology or plos computational biology or plos currents or plos genetics or "plos medicine public library of science" or plos neglected tropical diseases electronic resource or plos one electronic resource or plos pathogens).jn. (227024)

2 (bmc biochemistry or bmc bioinformatics or bmc biophysics or bmc biotechnology or bmc cell biology or bmc developmental biology or bmc ecology or bmc evolutionary biology or bmc genetics or bmc genomics or bmc immunology or bmc microbiology or bmc molecular biology or bmc neuroscience or bmc physiology or bmc plant biology or bmc structural biology or bmc systems biology or bmc veterinary research electronic resource).jn. (42964)

3 (bmc anesthesiology or bmc cancer or bmc cardiovascular disorders or bmc clinical pathology or bmc complementary & alternative medicine or bmc dermatology or bmc ear nose & throat disorders or bmc emergency medicine or bmc endocrine disorders or bmc family practice or bmc gastroenterology or bmc geriatrics or bmc health services research or bmc hematology or bmc infectious diseases or bmc international health & human rights or bmc medical education or bmc medical ethics or bmc medical genetics or bmc medical genomics electronic resource or bmc medical imaging or bmc medical informatics & decision making or bmc medical research methodology or bmc musculoskeletal disorders or bmc nephrology or bmc neurology or bmc nursing or bmc nutrition or bmc obesity or bmc ophthalmology or bmc oral health or bmc palliative care or bmc pediatrics or bmc pharmacology & toxicology or bmc pregnancy & childbirth or bmc psychiatry or bmc psychology or bmc public health or bmc pulmonary medicine or "bmc sports science medicine and rehabilitation" or bmc surgery or bmc urology or bmc womens health).jn. (76867)

4 1 or 2 or 3 (346855)

5 randomized controlled trial.pt. (457131)

6 controlled clinical trial.pt. (92290)

7 randomized.ab. (407065)

8 placebo.ab. (187639)

9 drug therapy.fs. (2005209)

10 randomly.ab. (287700)

11 trial.ab. (422799)

12 groups.ab. (1779227)

13 or/5-12 (4171773)

14 exp animals/ not humans.sh. (4440009)

15 13 not 14 (3605151)

16 4 and 15 (64362)

17 limit 16 to yr="2017" (8333)

3. **Search strategy for RCT studies published in 2016 in subscription journals:**

1 "new england journal of medicine".jn. (76372)

2 "journal of the american medical association".jn. (10839)

3 jama.jn. (70144)

4 "annals of internal medicine".jn. (31888)

5 lancet.jn. (133711)

6 or/1-5 (322954)

7 randomized controlled trial.pt. (462857)

8 controlled clinical trial.pt. (94069)

9 randomized.ab. (404436)

10 placebo.ab. (189133)

11 drug therapy.fs. (1994553)

12 randomly.ab. (280875)

13 trial.ab. (423516)

14 groups.ab. (1730004)

15 or/7-14 (4107184)

16 exp animals/ not humans.sh. (4401774)

17 15 not 16 (3551514)

18 6 and 17 (57391)

19 limit 18 to yr="2016" (1511)

4. **Search strategy for RCT studies published in 2017 in subscription journals:**

1 "new england journal of medicine".jn. (76150)

2 "journal of the american medical association".jn. (10837)

3 jama.jn. (70944)

4 "annals of internal medicine".jn. (32472)

5 lancet.jn. (134475)

6 or/1-5 (324878)

7 randomized controlled trial.pt. (457035)

8 controlled clinical trial.pt. (92285)

9 randomized.ab. (406922)

10 placebo.ab. (187596)

11 drug therapy.fs. (2004865)

12 randomly.ab. (287613)

13 trial.ab. (422641)

14 groups.ab. (1778615)

15 or/7-14 (4170619)

16 exp animals/ not humans.sh. (4439762)

17 15 not 16 (3604066)

18 6 and 17 (56718)

19 limit 18 to yr="2017" (818)
