# Supplementary material for: Completeness of reporting in abstracts of randomized controlled trials in subscription and open access journals: cross-sectional study
Source: Trials. 2019 Dec 2;20:669. doi: 10.1186/s13063-019-3781-x (PMC6889688; doi:10.1186/s13063-019-3781-x)
Supplement: Supplementary file 2 — Additional file 2. Reporting of CONSORT for Abstracts for individual journals. [file 13063_2019_3781_MOESM2_ESM.docx]

**Appendix 2. Adherence to CONSORT for Abstracts in individual journals**

**Table 1.** Adherence to CONSORT for Abstract checklist by articles published in *the* ***New England Journal of Medicine*** (n=63) in 2016-2017

| **Item** | Number of items reported (percent, 95% confidence interval) |
| --- | --- |
| Title | 2 (3%, 1-12%) |
| Trial design | 56 (89%, 78-95%) |
| **Methods** |  |
| Participants | 63 (100%, 94-100%) |
| Interventions | 63 (100%, 94-100%) |
| Objective | 47 (75%, 63-84%) |
| Outcome | 97 (98%, 89-99%) |
| Randomization | 3 (5%, 2-13%) |
| Blinding (masking) | 47 (75%, 63-84%) |
| **Results** |  |
| Numbers randomized | 11 (17%, 9-30%) |
| Recruitment | 63 (100%, 94-100%) |
| Numbers analysed | 17 (27%, 18-39%) |
| Outcome | 15 (24%, 15-36%) |
| Harms | 51 (81%, 70-89%) |
| **Conclusions** | 63 (100%, 94-100%) |
| Trial registration | 63 (100%, 94-100%) |
| Funding | 62 (98%, 90-100%) |

**Table 2.** Adherence to CONSORT for Abstract checklist by articles published in ***The Lancet*** (n=44) in 2016-2017

| **Item** | Number of items reported (percent, 95% confidence interval) |
| --- | --- |
| Title | 44 (100%, 92-100%) |
| Trial design | 44 (100%, 92-100%) |
| **Methods** |  |
| Participants | 44 (100%, 92-100%) |
| Interventions | 44 (100%, 92-100%) |
| Objective | 43 (98%, 88-100%) |
| Outcome | 43 (98%, 88-100%) |
| Randomization | 39 (87%, 76-95%) |
| Blinding (masking) | 44 (100%, 92-100%) |
| **Results** |  |
| Numbers randomized | 41 (93%, 82-98%) |
| Recruitment | 44 (100%, 92-100%) |
| Numbers analysed | 33 (75%, 61-85%) |
| Outcome | 26 (59%, 44-72%) |
| Harms | 41 (93%, 82-98%) |
| **Conclusions** | 44 (100%, 92-100%) |
| Trial registration | 44 (100%, 92-100%) |
| Funding | 44 (100%, 92-100%) |

**Table 3.** Adherence to CONSORT for Abstract checklist by articles published in ***JAMA*** (n=36) in 2016-2017

| **Item** | Number of items reported (percent, 95% confidence interval) |
| --- | --- |
| Title | 34 (94%, 80-99%) |
| Trial design | 35 (97%, 86-100%) |
| **Methods** |  |
| Participants | 36 (100%, 90-100%) |
| Interventions | 36 (100%, 90-100%) |
| Objective | 36 (98%, 90-100%) |
| Outcome | 36 (98%, 90-100%) |
| Randomization | 1 (3%, 0-14%) |
| Blinding (masking) | 35 (97%, 86-100%) |
| **Results** |  |
| Numbers randomized | 31 (86%, 71-94%) |
| Recruitment | 36 (100%, 90-100%) |
| Numbers analysed | 19 (53%, 37-68%) |
| Outcome | 23 (64%, 48-78%) |
| Harms | 18 (50%, 34-66%) |
| **Conclusions** | 36 (100%, 90-100%) |
| Trial registration | 36 (100%, 90-100%) |
| Funding | 0 (0%, 0-12%) |

**Table 4.** Adherence to CONSORT for Abstract checklist by articles published in ***Annals of Internal Medicine*** (n=6) in 2016-2017

| **Item** | Number of items reported (percent, 95% confidence interval) |
| --- | --- |
| Title | 6 (100%, 61-100%) |
| Trial design | 6 (100%, 61-100%) |
| **Methods** |  |
| Participants | 6 (100%, 61-100%) |
| Interventions | 6 (100%, 61-100%) |
| Objective | 6 (100%, 61-100%) |
| Outcome | 6 (100%, 61-100%) |
| Randomization | 2 (33%, 10-70%) |
| Blinding (masking) | 4 (67%, 30-90%) |
| **Results** |  |
| Numbers randomized | 4 (67%, 30-90%) |
| Recruitment | 6 (100%, 61-100%) |
| Numbers analysed | 2 (33%, 10-70%) |
| Outcome | 0 (0%, 0-39%) |
| Harms | 5 (83%, 44-97%) |
| **Conclusions** | 6 (100%, 61-100%) |
| Trial registration | 6 (100%, 61-100%) |
| Funding | 6 (100%, 61-100%) |

**Table 5.** Adherence to CONSORT for Abstract checklist by articles published in ***BioMedCentral series*** journals (n=56) in 2016-2017

| **Item** | Number of items reported (percent, 95% confidence interval) |
| --- | --- |
| Title | 43 (77%, 63-87%) |
| Trial design | 51 (91%, 80-97%) |
| **Methods** |  |
| Participants | 56 (100%, 94-100%) |
| Interventions | 55 (98%, 91-100%) |
| Objective | 55 (98%, 91-100%) |
| Outcome | 55 (98%, 91-100%) |
| Randomization | 2 (4%, 1-12%) |
| Blinding (masking) | 45 (80%, 68-87%) |
| **Results** |  |
| Numbers randomized | 28 (50%, 37-63%) |
| Recruitment | 55 (98%, 91-100%) |
| Numbers analysed | 11 (20%, 20-32%) |
| Outcome | 8 (14%, 7-26%) |
| Harms | 19 (34%, 23-47%) |
| **Conclusions** | 56 (100%, 94-100%) |
| Trial registration | 56 (100%, 94-100%) |
| Funding | 1 (2%, 0-9%) |

**Table 6.** Adherence to CONSORT for Abstract checklist by articles published in ***PLoS* journals** (n=63) in 2016-2017

| **Item** | Number of items reported (percent, 95% confidence interval) |
| --- | --- |
| Title | 52 (83%, 71-90%) |
| Trial design | 59 (94%, 85-96%) |
| **Methods** |  |
| Participants | 57 (90%, 81-96%) |
| Interventions | 60 (95%, 87-98%) |
| Objective | 60 (95%, 87-98%) |
| Outcome | 56 (89%, 79-95%) |
| Randomization | 2 (3%, 1-11%) |
| Blinding (masking) | 53 (84%, 73-91%) |
| **Results** |  |
| Numbers randomized | 25 (40%, 29-52%) |
| Recruitment | 62 (98%, 92-100%) |
| Numbers analysed | 18 (29%, 19-41%) |
| Outcome | 7 (11%, 5-21%) |
| Harms | 16 (25%, 16-37%) |
| **Conclusions** | 60 (95%, 87-98%) |
| Trial registration | 44 (70%, 58-80%) |
| Funding | 1 (2%, 0-8%) |
